# Virus persistence in pig herds led to successive reassortment events between swine and human influenza A viruses, resulting in the emergence of a novel triple-reassortant swine influenza virus

**DOI:** 10.1186/s13567-019-0699-y

**Published:** 2019-10-07

**Authors:** Amélie Chastagner, Emilie Bonin, Christelle Fablet, Stéphane Quéguiner, Edouard Hirchaud, Pierrick Lucas, Stéphane Gorin, Nicolas Barbier, Véronique Béven, Emmanuel Garin, Yannick Blanchard, Nicolas Rose, Séverine Hervé, Gaëlle Simon

**Affiliations:** 1 Swine Virology Immunology Unit, Ploufragan-Plouzané-Niort Laboratory, ANSES, BP53, 22440 Ploufragan, France; 2 Epidemiology, Health and Welfare Unit, Ploufragan-Plouzané-Niort Laboratory, ANSES, BP53, 22440 Ploufragan, France; 3 Viral Genetic and Biosecurity Unit, Ploufragan-Plouzané-Niort Laboratory, ANSES, BP53, 22440 Ploufragan, France; 4 Bretagne Loire University, Cité internationale, 1 place Paul Ricoeur, CS 54417, 35044 Rennes, France; 5 Animal Health Service, Coop de France, 43 Rue Sedaine, 75538 Paris cedex 11, France; 6 Operational Team, ESA Platform, 31 Avenue Garnier, 69007 Lyon, France; 7 Present Address: INRA, US 1426, GeT-PlaGe, 24 chemin de borde rouge - Auzeville, CS 52627, 31326 Castanet-Tolosan, France; 8 Present Address: GDS-France, 37 Rue de Lyon, 75012 Paris, France

## Abstract

This report describes the detection of a triple reassortant swine influenza A virus of H1_av_N2 subtype. It evolved from an avian-like swine H1_av_N1 that first acquired the N2 segment from a seasonal H3N2, then the M segment from a 2009 pandemic H1N1, in two reassortments estimated to have occurred 10 years apart. This study illustrates how recurrent influenza infections increase the co-infection risk and facilitate evolutionary jumps by successive gene exchanges. It recalls the importance of appropriate biosecurity measures inside holdings to limit virus persistence and interspecies transmissions, which both contribute to the emergence of new potentially zoonotic viruses.

## Introduction, methods, and results

Influenza A viruses (IAVs) are pathogens with high impact on public and animal health. Several mechanisms, including high mutation rate, reassortment of genes and host switch, are responsible for the genetic and antigenic evolution of IAVs [1]. The surveillance of swine IAVs (swIAVs) is of major concern to study IAV evolution in pigs and assess interspecies transmission risks. Indeed, pig could serve as an intermediate host for the adaptation of avian influenza viruses to mammals, as well as a host for the generation of reassortant viruses with genes of different origins, due to their susceptibility to both avian and human IAVs [1, 2]. Since the 2009 pandemic, four swIAVs lineages have become enzootic in the European pig population, i.e., avian-like swine H1N1 (H1_av_N1), pandemic-like swine H1N1 (H1N1pdm), human-like reassortant swine H1N2 (H1_hu_N2) and human-like reassortant swine H3N2 (H3N2), with relative frequencies varying from country to country [3, 4]. Reassortant viruses either with genes from different enzootic swIAVs or with gene(s) from enzootic swIAV combined to gene(s) from human seasonal IAV are occasionally detected in pigs [4, 5]. Whereas they are most of the time sporadically identified, such reassortants can adapt to the species and examples of novel circulating swIAVs have been evidenced locally in the recent years, such as in Denmark, Germany and United-Kingdom [4]. Recurrent influenza, i.e., swIAV infection in each successive batch of pigs reared, was suggested to be associated to swIAV enzootic persistence at the herd level, a situation that would favor co-circulation of different swIAV subtypes and/or co-infection events with enzootic swIAVs and human IAVs, both situations being a prerequisite to the emergence of novel reassortant viruses [6, 7].

This study reports the detection in France of a novel triple reassortant H1_av_N2 virus following two reassortment events that took place probably 10 years apart. This new reassortant has evolved from a swine H1_av_N1 virus that acquired, first the N2 segment of a seasonal human H3N2 virus, then the M segment of a H1N1pdm virus.

### Case description and preliminary investigations for IAV infections

In February 2010, epidemiological and microbiological investigations were implemented in a farrow-to-finish pig herd (farm A) located in the *Indre*-*et*-*Loire* administrative department (#37) in France, due to severe and repeated outbreaks of porcine respiratory disease complex. At that time, the herd counted 2100 sows and was managed with a 1-week batch interval. It purchased its breeding stock, and replacement gilts were housed in an acclimatization barn located on-site. First IAV infection was evidenced through serological analyses on fattening pigs. Hemagglutination inhibition (HI) assays, performed using a reference panel of antigens representative for European swine IAVs [3], revealed the presence of antibodies directed against the hemagglutinin (HA) of H1_av_N1 viruses known to circulate in French pig herds since the early 80’ [4]. In September 2011, after several months during which cough was reported in many batches of pigs from all physiological stages, the IAV genome was detected by M-gene RT-qPCR [8] in nasal swab supernatants taken on piglets of 7–8 weeks of age and exhibiting influenza-like illness (ILI) clinical signs, i.e., hyperthermia, apathy, dyspnea, sneezing and coughing for no more than 2–3 days for individual animals. Molecular subtyping using RT-qPCRs specifically designed for the amplification of the HA- and the neuraminidase (NA-) encoding genes from the different swIAVs circulating in France and in Europe [8] identified an HA gene from the H1_av_N1 lineage and a NA gene of N2 subtype, demonstrating an infection with a H1_av_N2 reassortant virus. In April 2012, a vaccination program was set up, consisting in the injection of sows at each breeding cycle, with one dose (2 mL/pig) of adjuvanted-inactivated trivalent (H1_av_N1, H3N2, H1_hu_N2) vaccine Gripovac^®^3 (Mérial, Lyon, France). However, the herd seemed to remain permanently infected as recurrent respiratory outbreaks continued to be reported by the farmer in successive batches of pigs, especially at the nursery stage. In October 2012, virus isolation from nasal swab supernatants taken on 7 week-old piglets led to the identification of a H1_av_N2 reassortant virus again. In 2013, the vaccination program was limited to gilts, with two injections (Gripovac^®^3) 3 weeks apart upon arrival in quarantine, followed by a vaccination booster 3 weeks before farrowing. In 2014, several batches of gilts were imported from Denmark and the number of sows increased to 2600. From March 2015, the herd opted for self-replacement of the breeding stock, and still ensuring quarantine of young breeding animals in the acclimatization building. However, the herd still experienced recurrent respiratory outbreaks, while the gilt vaccination program continued using the Respiporc FLU^®^3 vaccine (formerly Gripovac^®^3; IDT-Biologika, Dessau-Rosslau, Germany). In May 2016, the IAV genome was detected in nasal swabs taken on 7-week-old piglets with ILI of strong intensity, i.e., lasting longer than 4 days and including coughing fits. For the third time, molecular subtyping indicated an infection with a H1_av_N2 reassortant virus.

### Genetic characterization of the three H1_av_N2 reassortant viruses successively identified in 2011, 2012 and 2016 in farm A

**Figure 1:**
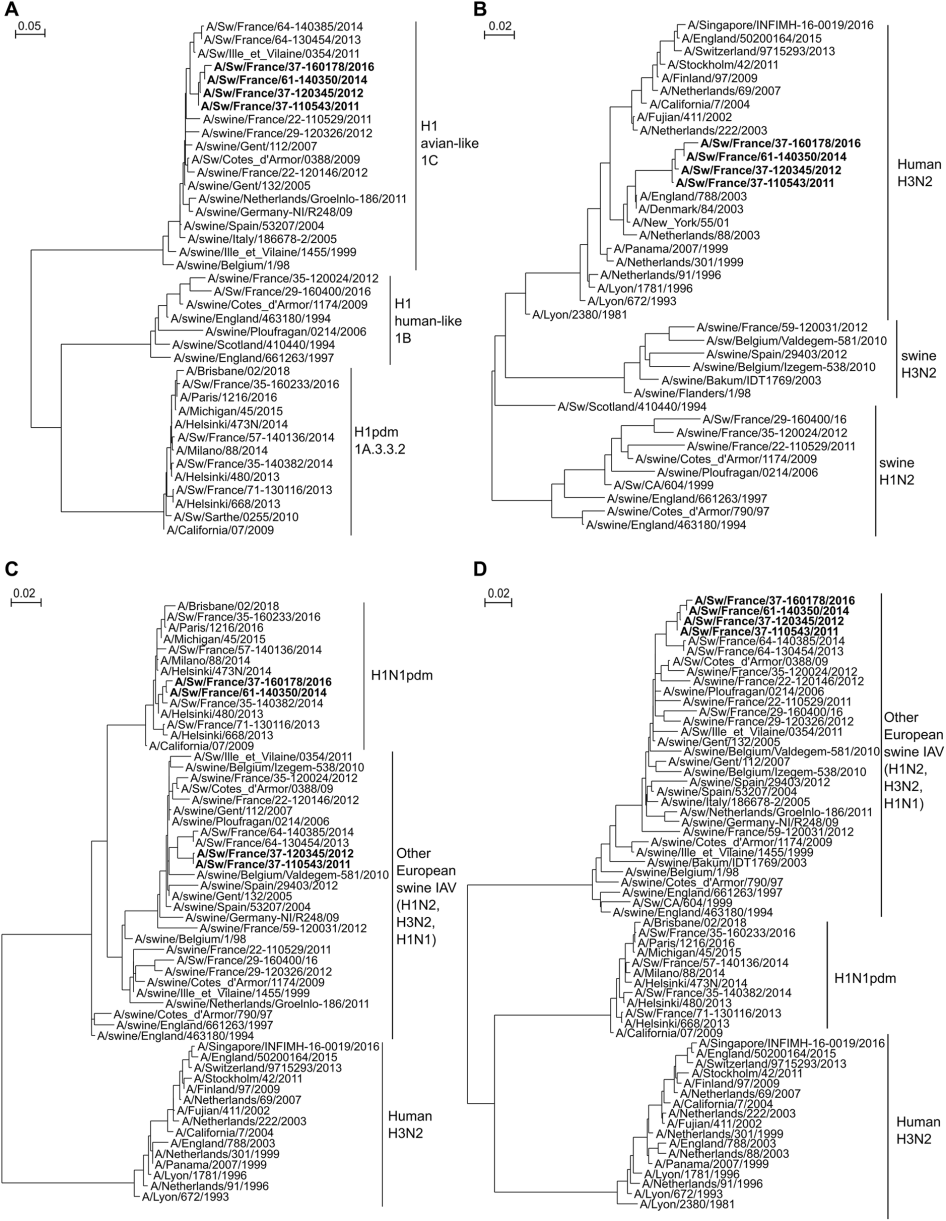
**Unrooted phylogenetic trees of European swine and human influenza A viruses. A** HA segment; **B** NA segment; **C** M segment; **D** Concatenation of the five other internal segments (PB2, PB1, PA, NP, NS). Phylogenetic trees were built with Seaview by maximum likelihood with the nucleotide substitution model HKY + G. The H1_av_N2 strains described in this study are indicated in bold. Only H1 and N2 subtypes were included in HA (**A**) and NA (**B**) trees, respectively.

The H1_av_N2 virus detected in 2011 and named A/Sw/France/37-110543/2011 did not propagate in cell culture or embryonated chicken eggs, probably due to limited amounts of virus particles in the 2011 nasal swab supernatants. However, sequencing was performed on viral RNA directly extracted from the swab sample. The H1_av_N2 viruses detected in 2012 and 2016 were propagated in MDCK cells and named A/Sw/France/37-120345/2012 and A/Sw/France/37-160178/2016, respectively. Whole genome sequences of these three virus strains were obtained by next generation sequencing (NGS) on an Ion Proton instrument (Life Technologies) as previously described [9]. The HA segments were confirmed to belong to the H1_av_ lineage (clade 1C.2.1, [10]) being closely similar to those of enzootic H1_av_N1 strains circulating in the French pig population (Figure 1A). By contrast, phylogenetic analyses showed that NA genes did not originate from enzootic swIAVs of the European H1_hu_N2 or H3N2 lineages, but were closely related (97.09% identity) to N2 genes of seasonal human H3N2 viruses isolated in 2003 (Figure 1B). The time of the most recent common ancestor (TMRCA) was estimated to 2003.528 [2003.094; 2003.801] using BEAST software, leading to the hypothesis that these reassortants originated from a co-infection with a H1_av_N1 swIAV and a 2003 human H3N2.

All internal genes of the three strains were shown to be very similar with each other and closely related to internal genes from H1_av_N1 and H1_hu_N2 viruses circulating in France, except the M-encoding gene of A/Sw/France/37-160178/2016 that belonged to the H1N1pdm lineage (Figures 1C and D and Additional file 1). The phylogenetic analysis indicated that this Mpdm segment was highly similar to that of H1N1pdm strains isolated in humans or pigs in 2013–2014 (TMRCA = 2013.863 [2013.492; 2014.216]), suggesting that a reassortment between the H1_av_N2 reassortant virus and a H1N1pdm virus took place in these years.

### Other H1_av_N2 reassortant viruses identified in France from 2010 to 2018

From February 2010 to December 2018, 18 other H1_av_N2 swIAVs were identified in French pig herds, leading the proportion of such reassortants to 2.22% (21/944) among the swIAVs identified in France thanks to diagnostic requests including those from Résavip, the national surveillance network for swIAV [11]. Thirteen of them were isolated on MDCK cells and sequence analyses revealed that only one harbored a N2 gene of human origin. This isolate, named A/Sw/France/61-140350/2014, was detected in June 2014 in 7-week-old piglets affected by ILI of normal intensity, in a post-weaning-finishing farm (farm B) located in the *Orne* administrative department (#61). Interestingly, it presented exactly the same genotype than A/Sw/France/37-160178/2016, with a 2003 seasonal N2 gene, a Mpdm segment from 2013 to 2014 and other genes from the H1_av_N1 lineage (Figure 1 and Additional file 1). Both viruses were 99.98–99.99% identical on all genes (data not shown). All other H1_av_N2 viruses shared H1_av_, N2 and internal genes from swine lineages (data not shown).

### Amino acid sequences and antigenic characterization of H1_av_N2 reassortant viruses

At the nucleotide level, phylogenetic analyses revealed that N2 genes of reassortant H1_av_N2 strains were closely related to those of 2003 human strains that did not derive from A/Fujan/411/2002(H3N2) as most of other H3N2 human strains isolated in 2003 worldwide but from the previous strain A/New York/55/01(H3N2) (Figure 1). Thus, deduced NA amino acid sequences of the four H1_av_N2 reassortants were compared to that of A/New York/55/01(H3N2) as a reference strain (Table 1). Alignments showed that all N2 glycoproteins exhibited several mutations in common, such as NA-K221N, NA-N329K and NA-S372L that, according to BII FluSurver, were described to be involved in antigenic drift of H3N2 human strains (Table 1). Looking for the occurrence of these three mutations in swine and human IAV N2 sequences retrieved from the Influenza Research Database, it appeared they were rarely observed in human strains from 2000 to 2005 regardless their genogroup (Table 1). By contrast, mutation S372L was observed in the majority of both recent human and swine strains, i.e., isolated from 2011 to 2016 (Table 1). Changing in NA-S372 residue was suspected to reduce the hemadsorption activity of human H3N2 and H2N2 strains [12, 13]. Interestingly, NA-K221N and two other mutations, i.e., NA-Y40H, and NA-V263I, were almost exclusively associated to swine but not human strains in the recent years 2011–2016 (Table 1) and could reflect divergent host-specific evolutions and/or swine host adaptation following inter-species transmission.

**Table 1:** **Amino acid differences in NA protein sequences of the H1**_**av**_ **N2 viruses described in this study as compared to NA residues in reference strain A/New York/55/01 (NY/55/01)**

Amino acid changes in swine H1_av_N2 strains					Reported effects^b^	Frequency of amino acid change in human or swine strains available in IRD^a^			
NY/55/01	A-11	A-12	B-14	A-16		Human 1957–1980 (*N* = 323)	Human 2000–2005 (*N* = 1921)	Human 2011–2016 (*N* = 8108)	Swine 2011–2016 (*N* = 4397)
T19	A	A	A	A	–	0.31	6.98	0.04	4.82
L23	F	F	F	F	–	0.31	47.11	98.87	5.53
A27	V	V	V	V	–	0	0.57	0.037	0.32
Y40	H	H	H	H	–	39.01	22.12	0.54	76.64
N47	T	T	T	I	–	0	0	0	1.07
A56	T	I	I	I	–	2.48(T)/97.21 (I)	97.19(T)/0.10 (I)	99.68(T)/0.19(I)	93.43(T)/4.21(I)
V66	–	–	M	M	–	0.31	0	0.11	0.86
E199	–	–	–	K	–	80.80	61.84	99.46	37.89
K221	N	N	N	N	Antigenic drift/escape mutant	99.69	1.04	0.78	81.49
K249	R	R	R	R	–	97.83	1.09	0.47	12.05
I254	V	V	V	V	–	0.31	0	0.30	1.02
V263	–	–	I	I	–	0.31	2.97	0.60	57.90
S271	N	N	N	N	–	0	0.10	0.16	0.23
Q273	H	H	H	H	–	0	0.05	0.01	0.07
N329	K	K	K	K	Antigenic drift/escape mutant, removes a potential *N*-glycosylation site	0	0.05	0.78	0.18
H336	R	–	–	–	–	0	0.05	0.04	0.45
D339	G	G	G	G	–	0	0.21	0.37	0.18
N358	H	H	H	H	–	0	0	0	0.07
S372	L	L	L	L	Antigenic drift/escape mutant	0.31	2.03	98.00	81.74
G401	–	–	D	D	–	70.59	2.76	0.42	10.98
E432	–	–	–	K	Antigenic drift/escape mutant	0	0.16	0.15	1.55
I469	M	M	M	M	–	0	0.10	0.04	3.78

Swine H1_av_N2 reassortants were abbreviated as following: A-11 for A/Sw/France/37-110543/2011 [Farm A], A-12 for A/Sw/France/37-120345/2012 [Farm A], B-14 for A/Sw/France/61-140350/2014 [Farm B] and A-16 for A/Sw/France/37-160178/2016 [Farm A].

^a^Frequency based on the occurrence of the mutated residue in N2 proteins from human or swine IAV strains whose sequences available in the Influenza Research Database on April, 16^th^ 2019.

^b^According to the BII FluSurver.

Comparison of H1_av_ protein sequences of H1_av_N2 strains to the HA sequence of the French H1_av_N1 ancestral strain A/Sw/Cotes d’Armor/0388/09(H1_av_N1) allowed identifying several mutations that were fixed on the H1_av_N2 reassortants (Table 2). According to BII FluSurver, many of them were reported to affect antigenic sites and suspected to be involved in antigenic drift (Table 2). Interestingly, the HA-G391R mutation into the HA2 subunit was never reported in swIAVs before, whereas described to increase the virulence of a human H1N1 prototype strain in MDCK cells and mouse lung [14]. More broadly, comparison of H1_av_ amino acid sequences to those of other H1_av_N1 and H1_av_N2 swIAVs did not permit the identification of mutations that would be specifically related to a maintenance of the HA/NA balance following the acquisition of a N2-encoding gene of human but not swine origin (data not shown).

**Table 2:** **Amino acid differences in HA protein sequences of the H1**_**av**_ **N2 viruses described in this study as compared to HA residues in reference strain A/Sw/Cotes d’Armor/0388/09 (CA/388/09)**

CA/388/09^a^	A-11	A-12	B-14	A-16	Reported effect^c^
V14	A^b^	A^b^	A^b^	A^b^	–
V22	I	I	I	I	–
S53	N	N	N	N	Antigenic drift/escape mutant
Q68	H	H	H	H	–
V74	–	–	I	I	–
L86	S	S	P	P	–
L88	–	–	–	P	Antigenic drift/escape mutant
S154	P	P	P	P	Antigenic drift/escape mutant
S156	–	–	L	L	Antigenic drift/escape mutant
G172	–	–	–	R	Antigenic drift/escape mutant and other
L178	–	–	–	I	–
K186	–	–	R	R	–
G187	–	–	–	E	–
I192	V^b^	V^b^	V^b^	V^b^	–
D202	–	–	V	V	–
T249	I	I	I	I	–
H270	–	–	–	Y	–
G277	S	S	S	S	Creates a new potential N-glycosylation site at position 275
V282	I^b^	I^b^	I^b^	I^b^	–
D286	N	N	N	N	–
H288	–	N	N	N	–
Y300	H^b^	H^b^	H^b^	H^b^	–
K304	–	–	N	N	–
S305	G	G	G	–	–
N306	S	S	S	S	–
E319	–	K	–	–	–
G391	R	R	R	R	Virulence
S393	N	N	N	N	–
I444	–	–	V	V	–
K460	R	R	R	R	–
E516	–	–	–	K	–

Swine H1_av_N2 reassortants were abbreviated A-11 for A/Sw/France/37-110543/2011 [Farm A], A-12 for A/Sw/France/37-120345/2012 [Farm A], B-14 for A/Sw/France/61-140350/2014 [Farm B] and A-16 for A/Sw/France/37-160178/2016 [Farm A].

^a^CA/388/09 is a reference strain representative of European swine avian-like H1_av_Ny viruses belonging to the HA clade 1C.2.1 (after Anderson et al. [10]).

^b^Residue fixed in French swIAV strains of avian-like H1 (H1_av_) lineage since 2000’.

^c^According to the BII FluSurver.

In order to further characterize the reassortants at the antigenic level, they were propagated on chicken embryonated eggs to be tested in HI tests using turkey’s red blood cells according to standard protocols [15]. Strains A/Sw/France/37-120345/2012, A/Sw/France/61-140350/2014 and A/Sw/France/37-160178/2016 were incubated with porcine hyperimmune sera containing antibodies directed against strains representative for the European enzootic swIAVs (Table 3). High HI titers (160–640) were obtained with antiserum to the H1_av_N1 reference strain, without any evidence of greater antigenic distance than reference strains (Table 3) or other contemporary H1_av_N1 strains (data not shown). Some cross-reactivity (HI titers 20–40) with antibodies directed against H1N1pdm reference strain was measured, as previously described for parental H1_av_N1 viruses [16].

**Table 3:** **Antigenic cross-reactivity between the novel H1**_**av**_ **N2 reassortant strains and reference strains representative of the European enzootic swIAV lineages**

Virus strain	Haemagglutination inhibition titer with hyperimmune sera against:			
	H1_av_N1 A/Sw/Cotes d’Armor/0388/09	H1_hu_N2 A/Sw/Scotland/410440/94	H3N2 A/Sw/Flanders/1/98	H1N1pdm A/Sw/Sarthe/0255/10
A/Sw/Cotes d’Armor/0388/09 (H1_av_N1)	1280	< 10	< 10	< 10
A/Sw/Scotland/410440/94 (H1_hu_N2)	< 10	1280	10	< 10
A/Sw/Flanders/1/98 (H3N2)	< 10	< 10	2560	< 10
A/Sw/Sarthe/0255/10 (H1N1pdm)	40	< 10	< 10	640
A/Sw/France/37-120345/12 (H1_av_N2)	320	< 10	< 10	20
A/Sw/France/61-140350/14 (H1_av_N2)	640	< 10	< 10	40
A/Sw/France/37-160178/16 (H1_av_N2)	160	< 10	< 10	< 10

## Discussion

In this study, we evidenced two successive reassortment events that probably occurred 10 years apart, i.e., around 2003 and 2013, respectively. Thus, a swine H1_av_N1 virus first acquired a N2 segment from a contemporary human H3N2 virus, and then a M segment from a H1N1pdm strain.

The human N2 incorporation into a French swine H1_av_N1 strain certainly resulted from a human-to-swine transmission of a human H3N2 virus around 2003, as similar N2 sequences were never described in European swine strains before. However, the origin of the parental human strain remains uncertain, as the few N2 sequences of 2000–2005 French H3N2 strains available in public databases do not belong to the A/New York/55/01(H3N2) lineage. The closest N2 genes that were retrieved belong to 2003 human H3N2 viruses isolated in England and Denmark, the latest originating from a reassortment between A/New York/55/01(H3N2) and A/Fujan/411/2002(H3N2) [17]. This first reassortant could have been introduced *in toto* in farm A between 2003 and 2010–2011, from another previously infected herd, or could have been generated in farm A itself thanks to a co-infection event. In any case, it is likely that this virus was maintained in farm A for some years, probably thanks to recurrent outbreaks that were shown to be associated with endemic persistence of swIAVs at the herd level [6]. These recurrent infections would be favored by herd management in batches, which introduces naïve individuals regularly in the herd [6]. Sow vaccination before farrowing was shown to reduce piglet susceptibility to swIAVs, thanks to maternally-derived antibodies. However, they did not prevent virus excretion by infected animals and extended virus spreading at the herd scale, a phenomenon that would also contribute to virus persistence on farms [18, 19]. It has also to be noted that other HxN2 swIAVs with N2 gene of human origin were previously reported to circulate in American, Italian and Danish herds for several years, confirming that this kind of reassortant virus could persist in swine populations [5, 20, 21].

Based on genetic similarity, it seemed unlikely that the triple reassortant virus was introduced *in toto* thanks to importations. The Mpdm segment acquired by the H1_av_N2 virus during the second reassortment could have been provided by a H1N1pdm virus, either subsequent to a human-to-swine transmission during the 2013–2014 seasonal epidemic or from a swine-to-swine transmission as this virus also circulates in pigs in France and other European countries [3, 9, 22]. What is obvious is that the Mpdm gene did not originate from a H1N1pdm strain belonging to the swine-divergent H1N1pdm lineage that was recently identified in pigs in France [9]. However, it cannot be excluded that the Mpdm gene was acquired from another unknown reassortant swIAV containing this genomic segment, as new viruses could be introduced in France thanks to importations of replacement sows for example. The second reassortment event might have occurred in farm A where the first reassortant virus was detected and shown to have persisted for months, possibly years, before. However, the isolation of a similar triple reassortant H1_av_N2 virus in farm B in 2014, at the time the second reassortment event was estimated to have occurred, poses questions about where this event took place. Phylogenetic distances between the four studied strains were congruent, exhibiting genetic evolution rates similar to those reported for swIAV strains across years [23]. This argues in favor of the hypothesis that the triple reassortant strains originated from a single source rather than from two independent reassortment events, which would have resulted in two H1_av_N2 triple reassortant strains exhibiting exactly the same gene constellation in the two distant farms. Based on farm A’s animal movement reports, any direct epidemiological link could be found between farms A and B, but a recent study showed that the French network of pig movements contributed to the dispersal of pathogens between non linked farms via node holdings that buy and sell pigs for all France [24]. Thus, it makes impossible to affirm that the second reassortment event took place in farm A and then the triple reassortant spread to farm B.

In any cases, the detection of such triple reassortant swIAVs in both farms A and B several years after the initial detection of the unusual parental reassortant virus in farm A gave evidence that reassortments are favored by swIAV persistence in pig herds, by co-circulation of swIAVs of different subtypes, and at last but not least, by transmission of IAVs from humans to pigs. Several examples of H1N1, H1N2 or H3N2 swIAVs containing the Mpdm gene were reported in other countries in Europe, Asia and the United States [4, 25, 26]. In the United States, such reassortants with Mpdm gene have been responsible for human infections at exhibition fairs since 2011 [26]. Whereas subsequent human-to-human transmission seemed limited, such zoonotic infections led WHO to anticipate in selecting vaccine strain candidates [27], taking into account that the Mpdm gene was demonstrated to increase intra- and/or inter-species transmission efficiency of IAV reassortants [28, 29]. Thus, the genomic constellation in addition to the specific mutations fixed incrementally in HA and NA glycoproteins after consecutive infectious cycles in pigs, could make the triple reassortant H1_av_N2 swIAVs at an increasing zoonotic risk as compared to parental viruses.

In conclusion, these events illustrate (i) the ability for swIAV to persist in a herd where recurrent influenza is made possible, (ii) the transmission of seasonal human IAV to pigs and (iii) subsequent co-circulations, co-infections and gene exchanges between swIAV(s) and/or human IAV, leading to the emergence of novel reassortant swIAV strains. That is why this study recalls the necessity to improve the management of influenza infections inside holdings to avoid swIAV persistence, e.g., by the export of consecutive piglet batches which was identified as the most efficient measure to limit recurrent swIAV infection in farrow-to-finish pig farms [30]. Finally, biosecurity measures should include actions aimed at reducing IAV interspecies transmissions, e.g., to limit the entry of ILI’s people, to encourage the use of protective mask and gloves, and to provide pig industry workers the annual influenza vaccine [22].

## Supplementary information

**Additional file 1. Scheme of genetic reassortment events that led to the detection of H1**_**av**_**N2 viruses in farm A and farm B between 2010 and 2016.** Donor viruses and viruses that resulted from reassortment events are illustrated above the timeline. The location (farm A or farm B) and year of isolation of the different virus strains that were sequenced in this study are marked with a black triangle below the timeline. The black circle indicate serological investigation and detection of antibodies (Ab) directed against an hemagglutinin from the H1_av_ lineage.

## Data Availability

All sequences obtained in this study are available in Genbank under Accession numbers KY364173-KY364180; KR700959-KR700966; MN326747-MN326754; MK943742-MK943749. Other datasets generated during and/or analyzed during the current study are available from the corresponding author on reasonable request.
