# Supplementary figures and images for: Virus persistence in pig herds led to successive reassortment events between swine and human influenza A viruses, resulting in the emergence of a novel triple-reassortant swine influenza virus
Source: Vet Res. 2019 Oct 7;50:77. doi: 10.1186/s13567-019-0699-y (PMC6781375; doi:10.1186/s13567-019-0699-y)

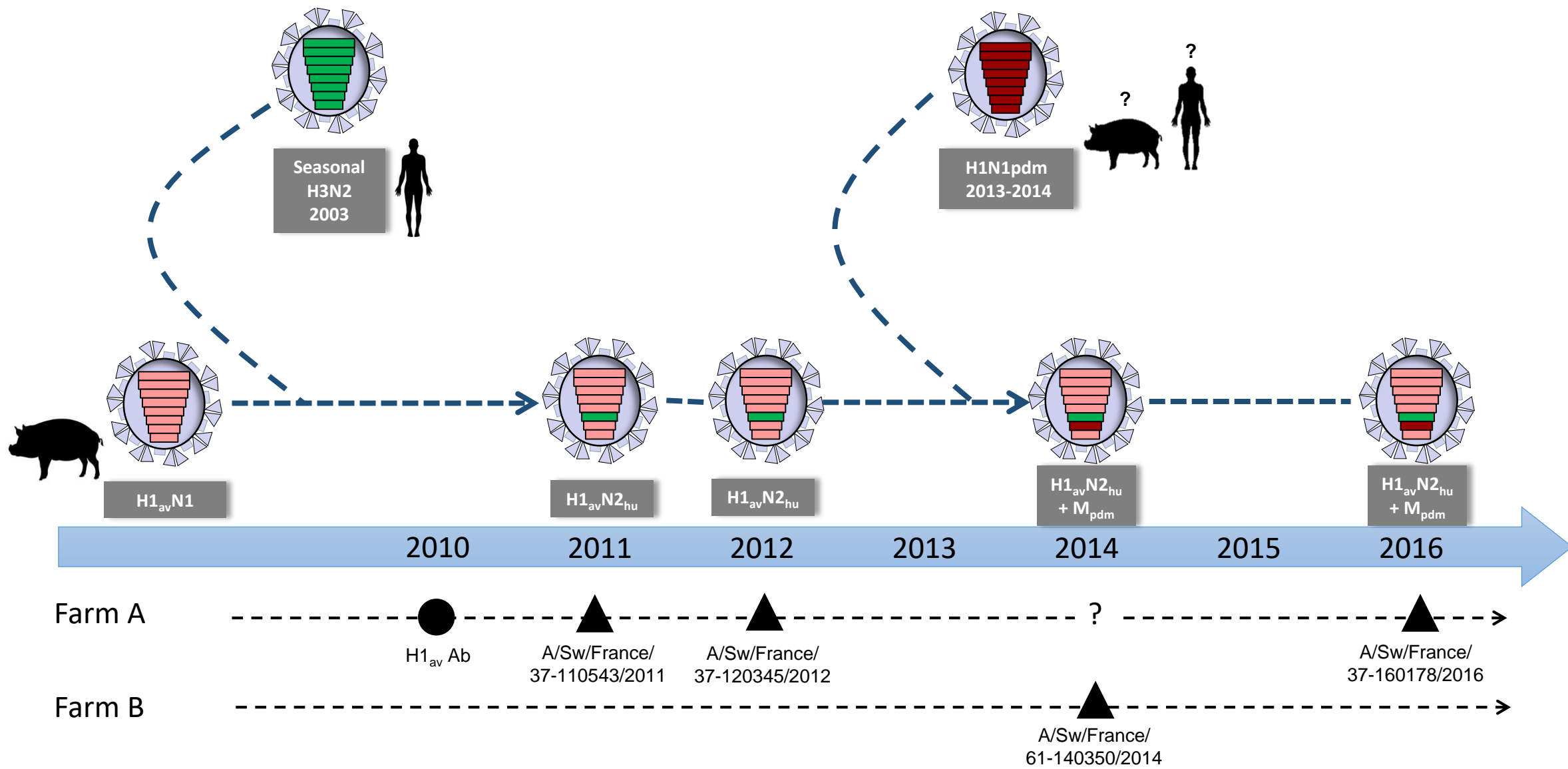

Supplement: Supplementary file 1 — Additional file 1. Scheme of genetic reassortment events that led to the detection of H1avN2 viruses in farm A and farm B between 2010 and 2016. Donor viruses and viruses that resulted from reassortment events are illustrated above the timeline. The location (farm A or farm B) and year of isolation of the different virus strains that were sequenced in this study are marked with a black triangle below the timeline. The black circle indicate serological investigation and detection of antibodies (Ab) directed against an hemagglutinin from the H1av lineage. [file 13567_2019_699_MOESM1_ESM.pdf]
